# The Effects of Transfusion upon the Heads of Decapitated Animals

**Published:** 1887-08

**Authors:** 


### THE EFFECTS OF TRANSFUSION UPON THE HEADS OF DECAPITATED ANIMALS.

In the *Journal* for March last, p. 194, mention was made of the results attained by MM. Hayem and Barrier in a series of experiments made upon the heads of animals recently removed from the body by sudden decapitation (as with the guillotine). The effects of transfusion of blood from a living animal into these detruncated heads were especially interesting and even startling; so much so indeed, that the authors of the memoire were induced to undertake a second series of experiments, more exhaustive than the first and conducted under more favorable circumstances. From their report, presented to the *Academie des Sciences* at a recent seance, we translate as follows:

"In the second series of experiments we adopted an arrangement which enabled us instantaneously, at a moment previously agreed upon and marked by a chronometer beating seconds, to pass a current of arterial blood from a living horse directly into the carotids of the detruncated heads of dogs. In this manner transfusion could be practiced before the head became inert, and at any moment during that period immediately following decollation, to which in our former report, we have given the name of the agonic period (*periode agonique.*) We had already found that to insure success it was necessary that the sanguineous irrigation should be abundant and sufficiently prolonged. These conditions were well met by the selection of a strong living horse as the source of blood supply.

The first question to the resolution of which we directed our experiments, was the determination of the latest point of time during the agonic period at which transfusion was capable of causing the reappearance of manifestations of consciousness and will-power. We found by experiment in this direction that a transfusion made ten seconds after decollation was perfectly successful in reproducing the evidence sought for; but that one made fifteen seconds afterward failed totally. We may therefore place the cessation of the period during which the re-animation of the encephalic centres, by means of oxygenated blood is possible, at from the eleventh to the fifteenth second after decollation.

The result must not be interpreted, however, to mean that will-power and consciousness last for the relatively long period of ten seconds after decapitation. On the contrary, it is our belief, based upon our former experiments, that decapitation produces an instantaneous or almost loss of sentinency and will-power—in other words a syncopal state. Our experiments simply prove that for the first ten seconds, at least, of this syncope, the anatomical elements of the sensor and excito-motor centres have the power of recovering their suspended faculties under the influence of arterial blood. As proof of the truth of this fact, we accept the phenomena produced during transfusion, viz: complex co-ordinate movement, either voluntary or provoked by the excitation of special senses.

Among the manifestations of this order we note as follows: movements of the ocular globes in their orbits, sometimes spontaneously and at others by the approach of a light, and even in response to the voice; the shaking of the entire head, produced by the contraction of the cervical muscles under the influence of fear or pain; the movement, by the same muscles, of the head from right to left or *vice versa*, as if in the effort to get away; contractions of the facial muscles giving the visage a marked expression of suffering and fear; attempts at lapping the tongue, made whenever the head was approached with a basin of water; efforts at swallowing or ejecting bits of sugar placed in the gullet; movements of the tongue in the effort to free itself from a bitter substance placed in contact with it, also in licking the lips; and finally, the emission, in certain cases, of plaintive cries when a current of air was forced up over the glottis from the cut end of the larynx.

When the transfusion was made within the first four seconds after decapitation, convulsive movements ceased at once; whereas, when undertaken at a later period the phenomena of contraction were persistent, particularly in the jaw and tongue. It is evident, therefore, that when the encephalic centres have undergone a period of total anæmia of several seconds duration, some of them remain in a state of hyperexcitability, in spite of transfusion.

When the limits of the first phase or stage have been passed we note a great change in the results of transfusion. The manifestations enumerated above no longer appear, but the influx of arterial blood still produces multiple movements of three descriptions, viz: convulsive, reflex and automatic.

To the first belong the movements of the lids and the muscles of the eyes (*nystagmus*) sometimes seen, the contiaction of the jaws and a powerful retraction of the tongue.

The reflexes are almost entirely ocular (palpebral and corneal, and a spontaneous winking). Cutaneous reflexes are extinct or on the point of becoming so. In the latter case they are confined to the lids, and consist simply in shutting them under an excitation of the nostrils. Here we may note the fact that the retraction of the tongue (referred to above as convulsive) may be markedly augmented by pinching the organ.

Finally the list is completed bv the establishment with greater or less frequency of automatic efforts at breathing.

Passing from the second phase, we enter upon the consideration of that which in our former report we have indicated as prolonging itself several minutes after the agonic period. In this we obtain only the palpebral and corneal reflexes, and automatic efforts at breathing.

The fourth and last phase manifests itself toward the tenth minute after decapitation. Transfusion prolonged beyond this point provokes a few incomplete efforts at inspiration, characterized by feeble movements of the nostrils and lips (without separation of the jaws) and accompanied by a scarcely perceptible retraction of the tongue. The ocular reflexes are gone, to return no more.

At the end of the twelfth minute the head becomes finally and forever inert. Transfusion can now produce fibrillary contractions only, and these are purely mechanical, due to the direct action of the column of blood upon the muscles.

We see from these experiments that the period during which it is possible to recall the activity of the cortical senso-motors by means of transfusion of arterial blood, is extremely short, covering not much longer than ten seconds after the act of decapitation. Further, we learn that the faculty of resuming a certain activity under the influence of living arterial blood, is lost in the encephalic centers from above downwards, progressively from the cortical layers to the bulbar foci, and that the last surviving of these centers—the *ultimum moriens*, is the inferior nucleus of the facial, and that at the end of the twelfth minute even this becomes definitely still and irresponsive to the action of the blood.".

In *La France Medicale* for March 29th there appears a further note from M. Laborde in relation to the first communication of MM. Hayem and Barrier (that first cited in the *Journal* and above referred to), concerning the experiments with the head of the guillotined criminal, Gangy. He says:

"In the interest of scientific exactness I should make the following simple rectification: It was in reality between the sixth and seventh minute after the decapitation that we received the head of the criminal Gagny. If I wrote in my recital 'toward the seventh minute' it was simply to take the extreme limit. There is a long difference between "seven minutes' and 'one hour,' which latter was the only period of time mentioned by MM. Hayem and Barrier in their recital of our transfusion experiments and experiences.

"Further than this—although from the nature of our arrangements it was impossible for me to make the essay at double transfusion until well toward the *twentieth* (20th) minute, nevertheless, when it was tried the results were well marked, and the phenomena of cerebral excitability persisted up to the fiftieth minute—or double the length of the period of their survival without transfusion. This result was expressly noted in our report, being the essential point of our conclusions and couched in these words: 'These new researches do not simply show that the post-mortal persistence of cerebral excitability is real and actual, but they demonstrate the possibility of doubling the period of this persistence by means of transfusion, and especially direct transfusion.'"

